# Associations between personality and whole‐brain functional connectivity at rest: Evidence across the adult lifespan

**DOI:** 10.1002/brb3.1515

**Published:** 2020-01-05

**Authors:** Sharon S. Simon, Eleanna Varangis, Yaakov Stern

**Affiliations:** ^1^ Cognitive Neuroscience Division Department of Neurology College of Physicians and Surgeons Columbia University New York NY USA

**Keywords:** aging, big five, connectivity, five‐factor model, functional magnetic resonance imaging, personality, resting‐state

## Abstract

**Introduction:**

Personality is associated with cognitive, emotional, and social functioning, and can play a role in age‐related cognitive decline and dementia risk; however, little is known about the brain dynamics underlying personality characteristics, and whether they are moderated by age.

**Methods:**

We investigated the associations between personality and resting‐state functional MRI data from 365 individuals across the adult lifespan (20–80 years). Participants completed the 50‐item International Personality Item Pool and a resting‐state imaging protocol on a 3T MRI scanner. Within‐network connectivity values were computed based on predefined networks. Regression analyzes were conducted in order to investigate personality–connectivity associations, as well as moderation by age. All models controlled for potential confounders (such as age, sex, education, IQ, and the other personality traits).

**Results:**

We found that openness was positively associated with connectivity in the default‐mode network, neuroticism was negatively associated with both the ventral and dorsal attention networks, and agreeableness was negatively associated with the dorsal attention network. In addition, age moderated the association between conscientiousness and the frontoparietal network, indicating that this association become stronger in older age.

**Conclusions:**

Our findings demonstrate that personality is associated with brain connectivity, which may contribute to identifying personality profiles that play a role in protection against or risk for age‐related brain changes and dementia.

## Graphical abstract

We investigated the associations between personality and resting‐state functional MRI data from 365 individuals across the adult lifespan (20–80 years). We found that openness was positively associated with connectivity in the default mode network, neuroticism was negatively associated with both the ventral and dorsal attention networks, and agreeableness was negatively associated with the dorsal attention network. In addition, age moderated the association between conscientiousness and the frontoparietal network, indicating that this association becomes stronger in older age.

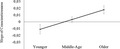

## 1 INTRODUCTION

There is increasing interest in understanding how personality may affect brain function and health across the adult lifespan. Personality consists of relatively stable patterns of behaviors, cognition, motivation, and emotional responses that characterize each individual. Currently, the five‐factor model (FFM) or “Big Five” is a widely accepted taxonomy of human personality that includes five traits: openness (i.e., the tendency to be imaginative, perceptive, curious, creative, and thoughtful), conscientiousness (i.e., the tendency to be organized, goal‐oriented, self‐disciplined, persistent, and suppress disruptive impulses), extraversion (i.e., the tendency to seek stimulation, be energetic, sociable, assertive, and active), agreeableness (i.e, the tendency to be cooperative, altruistic, and empathetic), and neuroticism (i.e., is the tendency to experience negative emotions, worries, and stress; Costa & McCrae, 1992; Goldberg, 1990; McCrae & Costa, 2003).

Although previous research stated that personality is relatively stable over time suggesting that personality becomes “set like plaster” by age 30 (i.e., the plaster hypothesis; Costa & McCrae, 1994, 1997; Costa, Metter, & Mccrae, 1994; Roberts & DelVecchio, 2000), there is compelling evidence showing changes in personality across adulthood, including in old age. Cross‐sectional and longitudinal studies indicate that aging is associated with lower levels of neuroticism, openness, and extraversion, and higher levels of agreeableness and conscientiousness (Allemand, Zimprich, & Hendriks, 2008; Caspi, Roberts, & Shiner, 2005; Costa & McCrae, 1997; Donnellan & Lucas, 2008; Helson, Jones, & Kwan, 2002; McCrae, Martin, & Costa, 2005; Roberts & Mroczek, 2008; Roberts, Walton, & Viechtbauer, 2006; Soubelet & Salthouse, 2011; Srivastava, John, Gosling, & Potter, 2003; Terracciano, McCrae, Brant, & Costa, 2005; Weiss et al., 2005).

Nevertheless, studies that incorporate a wider age range that includes older individuals (60 years and up) indicated that conscientiousness may present a curvilinear association with age, such that it increases up until middle age and decreases in older ages (Donnellan & Lucas, 2008; Terracciano et al., 2005). Besides the effect of age, it is important to consider that major life events can also affect personality traits and therefore be confounded with age since they occur in different phases of adult life. For instance, a large study found specific effects of major life events (i.e., first job, marriage, childbirth, separation, divorce, and retirement) on different personality traits (Specht, Egloff, & Schmukle, 2011).

Evidence indicates that personality traits, particularly conscientiousness, neuroticism, and openness, are associated with general health, longevity, cognitive performance (Chapman, Roberts, & Duberstein, 2011; Curtis, Windsor, & Soubelet, 2015), and dementia risk (Chapman et al., 2019; Terracciano & Sutin, 2019). Conscientiousness has been associated with reduced cognitive decline, while neuroticism has been associated with greater decline (Caselli et al., 2016; Hock et al., 2014; Luchetti, Terracciano, Stephan, & Sutin, 2016). Similarly, individuals who scored lower on conscientiousness and higher on neuroticism shower greater risk for development of Alzheimer's Disease (AD; Terracciano et al., 2014). Critically, these traits are also associated with dementia risk factors (Curtis et al., 2015; Terracciano et al., 2014). For instance, conscientiousness has been negatively associated with cigarette smoking, physical inactivity, obesity, and diabetes, while neuroticism is associated with higher risk for psychopathology, especially anxiety disorders and depression (Lahey, 2009). It has been hypothesized that higher openness can be a protective factor in the contact of cognitive aging since it is associated with greater participation in a cognitively enriching lifestyle; however, there are inconsistencies in studies that have explored this relationship. Studies found that openness did not predict differences in cognitive trajectories over time (Caselli et al., 2016; Sharp, Reynolds, Pedersen, & Gatz, 2010), while others found openness was associated with better cognitive performance and less decline over the time (Chapman et al., 2012; Luchetti et al., 2016). In addition, higher openness and agreeableness have been associated with a slightly reduced risk of AD, but not extraversion (Terracciano et al., 2014), although in another study some association has been found between higher extraversion and steeper decline (Chapman et al., 2012).

Despite the above evidence showing the influence of personality on general health, cognitive aging, and dementia risk, it is still unclear as to what possible mechanisms underlying these relationships might be. A better understanding of the effect of personality on brain health metrics across adulthood can advance this area of research to gain a more complete understanding of how personality may affect cognitive health in aging. Resting‐state functional connectivity (RSFC) is considered a viable approach to capture the complex intrinsic neural architecture underlying personality (Nostro et al., 2018) and can describe personality differences in terms of networks dynamics (Toschi, Riccelli, Indovina, Terracciano, & Passamonti, 2018). Specifically, RSFC is based on resting‐state functional magnetic resonance imaging (RS‐fMRI), which measures spontaneous low‐frequency fluctuations in blood oxygen level‐dependent (BOLD) signal in subjects at rest. RS‐fMRI has attracted attention for its ability to measure correlations in neural activity (via BOLD signal) between brain regions, regardless of their spatial proximity (Power et al., 2011; Schaefer et al., 2018), in order to identify co‐activation patterns among regions (i.e., networks).

There are several brain networks that have been established to have specific cognitive implications that replicate across independent adult samples (Power et al., 2011), such as the default‐mode network (DMN; associated with self‐reflection and mind‐wandering thought; Buckner, Andrews‐Hanna, & Schacter, 2008; Raichle et al., 2001), ventral and dorsal attention networks (VAN and DAN, respectively; associated with dynamic attentional control; Corbetta & Shulman, 2002; Fox, Corbetta, Snyder, Vincent, & Raichle, 2006; Vossel, Geng, & Fink, 2014), salience network (SAN; associated with cognitive control; Seeley et al., 2007), frontoparietal network (FPN; associated with executive function; Power et al., 2011), and cingulo‐opercular network (CON; associated with alertness; Dosenbach et al., 2007; Power et al., 2011). Past studies consider these networks to be cognitive or associative networks (Chan, Park, Savalia, Petersen, & Wig, 2014; Geerligs, Renken, Saliasi, Maurits, & Lorist, 2015), since the regions implicated within these networks tend to correspond to areas of task‐related activation on relevant cognitive tasks (Barch, 2017). Despite this, the relationship between these brain networks and personality is still poorly understood.

Regarding openness/intellect, is suggested that DMN play a relevant role given it implications on imagination, imagery, and creativity (Allen & DeYoung, 2016; Beaty et al., 2014). For instance, higher levels of openness/intellect were associated with higher FC between DMN and regions or networks associated with cognitive control (Adelstein et al., 2011; Beaty et al., 2018), and predicted global efficiency of a network comprised of DMN nodes (Beaty et al., 2016). In relation to conscientiousness, FPN and VAN have been considered good candidates for a neural substrate since they are relevant to reorienting attention away from distractions and toward stimuli important for goal pursuit (Allen & DeYoung, 2016). In this vein, a large study (*N* = 818) found that only conscientiousness was linked to measures of higher FC in the FPN and DMN (Toschi et al., 2018). In addition, a recent study found that conscientiousness was positively associated with within‐network connectivity of the “goal priority network,” a broad neural network incorporating VAN and SAN regions (Rueter, Abram, MacDonald, Rustichini, & DeYoung, 2018).

It has been hypothesized that individuals with higher levels of neuroticism present deficits in emotional and self‐regulation (Robinson, Ode, Wilkowski, & Amodio, 2007; Tamir, 2005), which could suggest reduced connectivity between the amygdala and frontal regions (Allen & DeYoung, 2016). For instance, higher levels of neuroticism were associated with lower FC between the amygdala and the dorsomedial prefrontal cortex, but also temporal regions such as middle temporal gyrus and temporal pole (Adelstein et al., 2011; Aghajani et al., 2014). In addition, there is evidence that neuroticism is associated with overall weaker FC when considering a brain‐wide network (Nostro et al., 2018), FPN, or DMN, and stronger FC in the networks implicated in emotional processing and negative affect (i.e., “affective” network and CON; Servaas et al., 2015). These findings are in line with the “mental noise hypothesis,” which states that higher neuroticism is characterized by increased mental noise, contributing to variability in cognitive performance and cognitive deficits, particularly in cognitive control and attention (Bredemeier, Berenbaum, Most, & Simons, 2011; Robinson & Tamir, 2005; Robison, Gath, & Unsworth, 2017). In addition, some of the negative associations between neuroticism and brain connectivity are consistent with the networks implicated in major depression (e.g., DMN, DAN and “cognitive control” network; Yan et al., 2019; Yao et al., 2019) and anxiety (e.g., VAN, FPN, DMN, CON; Sylvester et al., 2012).

Extraversion has not being associated with RSFC in studies using a whole‐brain approach (Dubois, Galdi, Han, Paul, & Adolphs, 2018; Toschi et al., 2018). Despite that, it has been associated with strengthened FC between different ROIs (e.g., amygdala, ACC, and precuneus) and regions involved in the reward system (Adelstein et al., 2011; Passamonti et al., 2015), and emotional and motivational processing (Aghajani et al., 2014). There is no evidence that Agreeableness is associated with RSFC in studies using a whole‐brain approach (Dubois et al., 2018; Toschi et al., 2018). Nevertheless, agreeableness is associated with greater processing of social information and is likely to be involved in emotional regulation and the ability to suppress aggressive impulses (Allen & DeYoung, 2016). For instance, resting‐state studies found that higher levels of agreeableness are associated with higher FC between ROIs (ACC and precuneus) and regions involved in empathy and social information processing (Adelstein et al., 2011); and between posterior cingulate cortex and DMN regions (Ryan, Sheu, & Gianaros, 2011). In addition, a meta‐analysis found a common disrupted cognitive control network across aggressive individuals with psychiatric diagnoses (Wong et al., 2019), suggesting that networks related to cognitive control may be associated with agreeableness.

In summary, evidence from past studies indicates that personality is associated with different aspects of RSFC; however, these studies vary in terms of approach (e.g., seed‐based vs. whole‐brain), and network definitions, which limits generalizable conclusions and contributes to variability in the results. In addition, several studies considered sex, and sometimes age, as control variable, but most failed to control for intelligence and/or education, factors that may influence both personality and brain function. Critically, all RSFC studies reviewed above included only young adults, indicating a gap in the literature considering an older population, remaining unclear whether age can moderate personality–connectivity associations. This is a relevant aspect since age can affect both personality (Donnellan & Lucas, 2008; Terracciano et al., 2005) and RSFC (Chan et al., 2014; Geerligs et al., 2015). In addition, understanding how personality is associated with brain functioning in healthy individuals across adulthood, and whether this association differs as a function of age can advance the understanding of personality's role as a protective factor against age‐related cognitive decline and dementia.

### 1.1 The present study

Taking into account the aforementioned literature and methodological considerations, the aims of the current study were twofold. First, to extend previous research by examining the association between personality and RSFC, considering a number of methodological advantages: (a) a relatively large cohort (*N* = 365); (b) a wide age range representing the adult lifespan (20–80 years); (c) control of variables that may affect personality and/or brain function, such as age, sex, education, and IQ; and (d) using predefined networks considered to be stable across replication (Power et al., 2011). Considering this last item, we focused on networks that are thought to be relevant for cognitive and/or emotional processing (i.e., CON, DAN, DMN, FPN, SAN, and VAN) and therefore critical to investigate the underlying brain dynamics of personality. Second, to investigate whether age moderates any associations between personality traits and within‐network connectivity. To the best of our knowledge, this is the first study to address this question including a sample with a wide age range representing the adult lifespan. Understanding whether age plays a role in the relationship between personality and functional connectivity may elucidate underlying mechanisms suggesting personality characteristics that may be protective against or a source of vulnerability for age‐related cognitive decline and dementia.

Regarding our first aim, based on the previous literature, it is hypothesized that some personality traits will be associated with within‐network connectivity. In brief, higher levels of openness will be associated with stronger within‐DMN connectivity; higher levels of conscientiousness would be associated with higher within‐network connectivity in networks related to cognitive control and goal priority, possibly FPN, CON, VAN, and SAN; and higher levels of neuroticism would be associated with weaker within‐network connectivity in networks relevant for executive functions and attention control such as FPN, VAN, and DAN, as well as emotional regulation, such as the DMN. We did not predict robust associations between RSFC and extraversion or agreeableness. Regarding the second aim, we predicted that age would moderate personality–RSFC relationships; however, we consider this to be an exploratory aim since there is no previous literature to support specific hypotheses.

## 2 MATERIALS AND METHODS

### 2.1 Participants

A total of 365 participants were included in the present study (age range: 20–80 years). The sample was comprised of participants who completed the baseline visit for either of two studies: the Reference Ability Neural Network (RANN) study or Cognitive Reserve (CR) study (Habeck et al., 2016; Stern, 2009; Stern et al., 2014). Both studies included the same inclusion/exclusion criteria, the same structural and resting‐state functional imaging protocols, and most of the same cognitive assessments and questionnaires. The primary difference between the two studies was the functional task‐based imaging protocols used, which will not be analyzed in the current study.

Participants were recruited using established random market mailing procedures, and written informed consent was obtained from all participants prior to study participation. All participants were native English speakers, right‐handed, free of MRI contraindications, and read at a fourth grade reading level or above. Screening was performed prior to enrollment to ensure that no participants had any psychological or medical conditions that could affect cognitive functioning. Specifically, for older adults, performance on our neuropsychological battery (details below) that was indicative of mild cognitive impairment or dementia was grounds for exclusion. Global cognitive functioning was assessed with the Mattis Dementia Rating Scale (Lucas et al., 1998), on which a minimum score of 134 was required for inclusion in the study. Additionally, in order to be included in the present analyses participants had to have personality data, complete a resting‐state scan protocol, and have less than 30% motion artifact data removal (scrubbing) from that resting‐state scan (Parkes, Fulcher, Yucel, & Fornito, 2018; Power, Barnes, Snyder, Schlaggar, & Petersen, 2012).

### 2.2 Behavioral data

#### 2.2.1 Neuropsychological assessment

Participants completed an extended neuropsychological battery. Similar to previous research (Salthouse et al., 2015; Soubelet & Salthouse, 2011), we created four composite cognitive domain scores based on performance on several cognitive tests: *Reasoning*: Wechsler Adult Intelligence Scale (WAIS‐III) Matrix Reasoning, Letter‐Number Sequencing, and Block Design tests (Wechsler, 1997). *Vocabulary*: WAIS‐III Vocabulary test, the Wechsler Test of Adult Reading (WTAR; Wechsler, 2001), and the American National Adult Reading Test (AMNART; Grober, Sliwinski, & Korey, 1991), *Memory*: Selective Reminding Test (SRT); last trial, continuous long‐term retrieval, and last retrieval (Buschke & Fuld, 1974), and *Speed of Processing*: WAIS‐III Digit Symbol, Stroop Color Naming test (Golden, 1975), and Trail Making Test (TMT)‐A (time) (Reitan, 1978). Following collection of all neuropsychological task data, performance on each task was *z*‐scored relative to the mean and standard deviation for each task within the whole sample of participants enrolled in the RANN and CR studies who completed these assessments. The *z*‐scores for all tasks within each cognitive domain were then averaged in order to generate domain‐based *z*‐scores.

#### 2.2.2 Personality

Personality traits were measured using the 50‐item version of the International Personality Item Pool (IPIP), to evaluate five major dimensions of personality based on the five‐factor model: openness, conscientiousness, extraversion, agreeableness, and neuroticism (reversed emotional stability; Goldberg, 1999). Participants rated themselves on a 5‐point scale ranging from “Strongly Agree” to “Strongly Disagree” with respect to how well each statement described them.

### 2.3 Neuroimaging data

#### 2.3.1 fMRI scan parameters

Neuroimaging data were collected during a 5‐(*n* = 124) or 9.5‐(*n* = 241) minute resting‐state fMRI protocol. All participants completed these scans on a 3T Philips Achieva Magnet. T1‐weighted images of the whole brain were acquired for each subject with a Magnetization Prepared Rapid Gradient Echo (MPRAGE) sequence with the following parameters: TE/TR: 3/6.5 ms; Field of view: 256 mm; Flip angle: 8°; In‐plane resolution: 256 × 256 voxels; Slice thickness/gap: 1/0 mm; and Slices: 180. fMRI blood oxygen level‐dependent (BOLD) resting‐state scans were collected with the following parameters: TE/TR: 20/2,000 ms; Flip angle: 72°; In‐plane resolution: 112 × 112 voxels; Slice thickness/gap: 3/0 mm; and Slices: 37.

#### 2.3.2 fMRI data processing

Images were preprocessed using an in‐house developed native space method (Razlighi et al., 2014) as described previously (Varangis, Habeck, Razlighi, & Stern, 2019). The preprocessing pipeline included slice‐timing correction and motion correction performed in FSL (Jenkinson, Bannister, Brady, & Smith, 2002; Jenkinson et al., 2012), calculation of frame‐wise displacement (FWD; as described in Power et al., 2011), volume replacement for contaminated volumes (Carp, 2013), band‐pass filtering using flsmaths–bptf (Jenkinson et al., 2012), and residualization of the processed data with respect to FWD, root mean square difference of the BOLD signal, left and right hemisphere white matter, and lateral ventricular signals (Birn, Diamond, Smith, & Bandettini, 2006). T1 image segmentation was performed using FreeSurfer (Dale, Fischl, & Sereno, 1999, Fischl et al., 2002, Fischl et al., 2004) and inspected visually for any inaccuracies. In order to perform the functional connectivity analyses described below, the coordinates of the 264 ROIs identified by Power et al. (2011) were transferred to native space via nonlinear registration of the subject's structural scan to the MNI template using the ANTS software package. Next, a 10‐mm‐radius spherical mask was generated for each coordinate and intersected with the FreeSurfer gray matter mask in order to derive the gray matter‐registered ROI masks for each of the 264 ROIs. An intermodal, intra‐subject, rigid‐body registration of the fMRI reference image and T1 scan was then performed using FLIRT with six degrees of freedom, normalized mutual information as the cost function (Jenkinson & Smith, 2001), in order to transfer ROI masks from T1 space to fMRI space. These transferred ROI masks were used to average all voxels within each mask to obtain a single fMRI time series for each of the 264 ROIs.

Time‐series data from each ROI were used to generate correlation matrices among all ROIs (264 ROIs by 264 ROIs) and were then *z*‐transformed to generate normalized correlation matrices for each participant. The diagonal of each correlation matrix was set to “NA” for all analyses, in order to remove correlations between an area and itself from analyses. ROIs were then labeled based on the Power (2011) network assignments, with the following six networks being selected for analysis based on their inclusion as cognitive or “association” networks in past studies (Chan et al., 2014); default mode (DMN; 58 ROIs), salience (Sal; 18 ROIs), cingulo‐opercular (CO; 14 ROIs), frontoparietal (FP; 25 ROIs), dorsal attention (DAN; 11 ROIs), and ventral attention (VAN; 9 ROIs).

#### 2.3.3 Functional connectivity analyses

**Figure 1:**
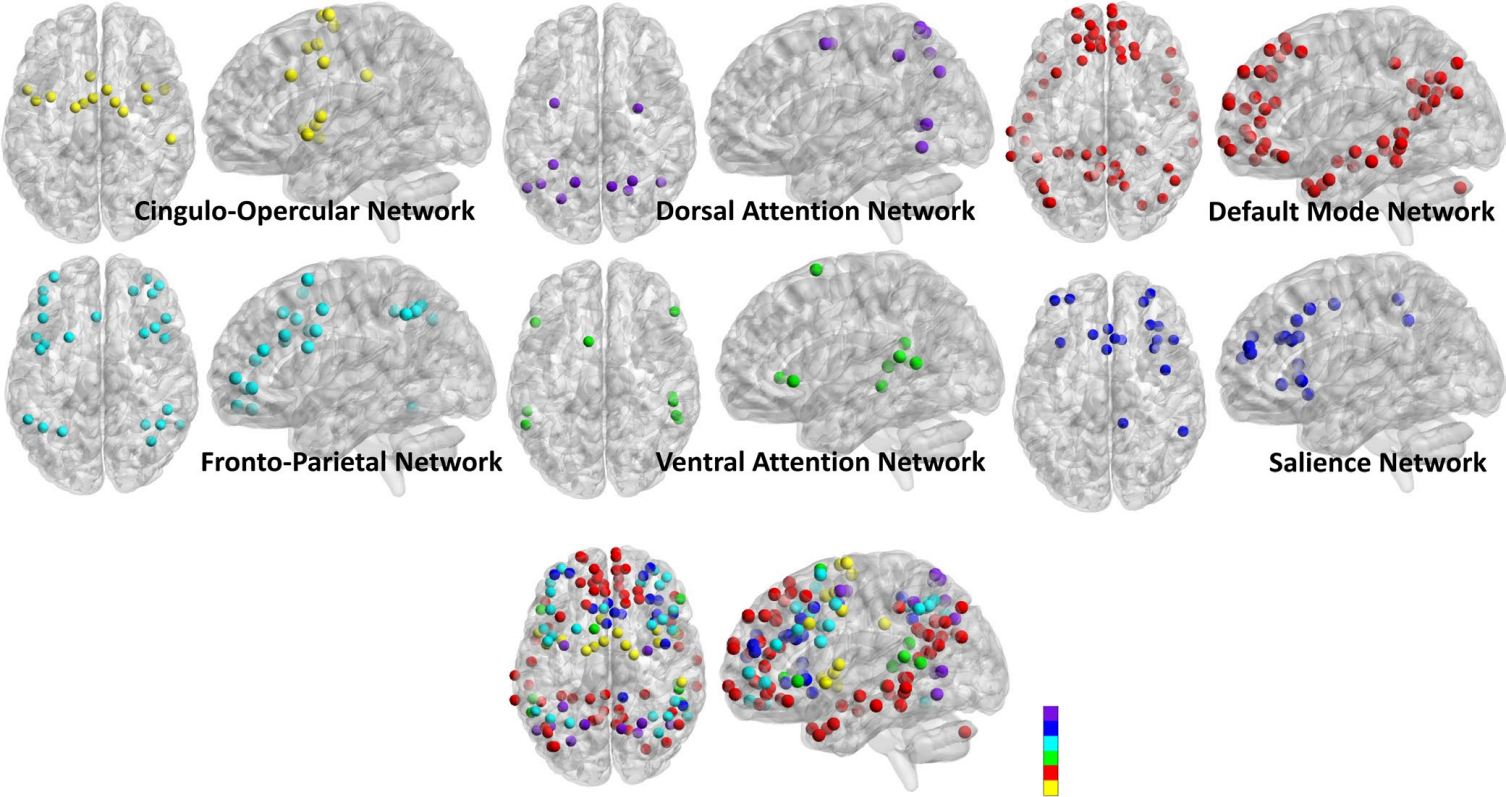
Regions of interest (ROIs) reflecting each cognitive network. *Note:* Regions of interest (ROIs) making up each of the six cognitive networks were superimposed on a standard 3D brain template. Images were generated using BrainNet Viewer by creating spheres around each of the Power (2011) ROI coordinates corresponding to the networks used in the present analysis

Individual *z*‐transformed correlation matrices were used to compute the measures of functional connectivity. The average positive within‐network correlation was computed by setting all negative correlation values to “NA” and then taking the average within‐network positive correlation for each of the six cognitive networks mentioned above, as in previous work by our group (Varangis, Habeck, et al., 2019). Although it is possible to include negative correlations in a network analysis (Rubinov & Sporns, 2011), because of the ambiguity regarding the meaning of negative correlations (Chai, Castanon, Ongur, & Whitfield‐Gabrieli, 2012; Chan et al., 2014; Murphy, Birn, Handwerker, Jones, & Bandettini, 2009; Scholvinck, Maier, Ye, Duyn, & Leopold, 2010), all negative correlations in participants’ correlation matrices were excluded from analysis. Within‐network correlations were characterized as those reflecting correlations among ROIs within a specific network; thus, the data for this analysis included the average within‐network positive correlation (six values; one average within‐network positive correlation computed per network) for each participant. In addition, for illustration purposes, regions of interest (ROIs) on each network were superimposed on a standard 3D brain template making up each of the six cognitive networks (Figure 1). The images were generated using BrainNet Viewer (Xia, Wang, & He, 2013—http://www.nitrc.org/projects/bnv/) by creating spheres around each of the Power (2011) ROI coordinates corresponding to the networks used in the present analysis.

#### 2.3.4 Statistical analyses

In order to examine relationships between personality and within‐network connectivity metrics, multiple regression models were computed using connectivity within each of the networks as a dependent variable. In Model 1, we investigated the effect of each of the personality traits on each of the average within‐network correlations. We ran separate models for each network (dependent variable: within‐network connectivity): We first entered demographic variables that may affect personality or connectivity values (i.e., age and sex) and then entered all personality variables in order to assess the unique effect of each personality trait on each within‐network connectivity metric. In Model 2, we added two‐way interaction terms between age and each personality variable to the previous model in order to examine whether age could moderate the association between personality and each network. Of note, the interaction terms were calculated using mean‐centered variables and were entered in separate models for each network. To more closely understand the nature of the interaction, we tested conditional effects of age moderation considering three age groups, which we labeled as Younger (36.8 yo; −1 *SD*), Middle Age (53,4 yo; mean), and Older (70.1; +1 *SD*). Conditional effects of moderation were examined with PROCESS (Hayes, 2012), and analyses were performed using SPSS 22 (SPSS).

## 3 RESULTS

### 3.1 Demographics and descriptive statistics

Participant demographics are described in Table 1. The mean age of participants was 53.4 years (range of 20–80 years), and mean education was 16.1 years (range of 9–24 years). There were no differences in sex and education when stratifying by age groups. Personality scores revealed only neuroticism differed as a function of age, indicating lower neuroticism in older adults. In addition, cognitive performance reflected the expected differences across the age groups, indicating that older participants show higher verbal IQ and vocabulary scores, but lower scores on tasks of reasoning, episodic memory, and processing speed.

**Table 1:** Demographics and cognitive characteristics

	All	Young	Middle age	Older adults	*p*‐value
Demographics	20–80 years	20–39 years	40–59 years	60–80 years	
Number of subjects	365	96	88	181	
Age, *M* (*SD*), years	53.4 (16.6)	29.5 (4.9)	50.6 (5.4)	67.5 (5.1)	<.001*
Sex, % of Women	55.1%	51.0%	61.4%	54.1%	.35
Education, *M*(*SD*), years	16.1 (2.3)	15.8 (2.4)	16.1 (2.3)	16.3 (2.3)	.16
Personality					
Openness	0.004 (1.0)	0.11 (1.0)	−0.01 (1.0)	−0.04 (0.99)	.46
Conscientiousness	−0.02 (1.0)	−0.06 (0.99)	0.04 (1.0)	−0.03 (0.97)	.73
Extraversion	−0.001 (1.0)	0.004 (1.1)	0.02 (0.95)	−0.01 (0.98)	.93
Agreeableness	−0.02 (1.0)	−0.08 (1.0)	−0.04 (1.1)	0.02 (0.93)	.65
Neuroticism	0.006 (0.99)	0.24 (1.1)	0.01 (1.0)	−0.12 (0.91)	.01*
Cognition					
IQ Scores^a^	117.0 (8.5)	113.4 (8.3)	115.9 (8.1)	119.4 (8.1)	<.001*
Vocabulary score	0.02 (0.9)	−0.32 (0.9)	−0.07 (0.8)	0.26 (0.8)	<.001*
Reasoning score	0.07 (0.8)	0.62 (0.7)	−0.05 (0.8)	−0.16 (0.7)	<.001*
Memory score^b^	0.05 (0.9)	0.60 (0.7)	0.13 (0.9)	−0.27 (0.8)	<.001*
Speed score^c^	0.02 (0.8)	0.72 (0.7)	0.05 (0.7)	−0.35 (0.6)	<.001*

a Verbal IQ scores are based on American National Reading Test (AMNART).

b There are missing data for two subjects, and scores reflect 363 subjects.

c Lower values of Speed scores reflect worse (slower) performance. Personality and cognitive scores are represented by *z*‐scores.

* Significant *p*‐values (<.05).

### 3.2 Relationships between personality and within‐network connectivity

Regression model results for the effects of the personality traits on each within‐network connectivity metric are presented in Table 2. The models were run separately for each network and controlled for age, sex, education, and IQ**.** The regression parameter for openness was found to be significant in predicting within‐DMN connectivity (β = .14, *p* = .02) and the regression parameter for neuroticism significantly negatively predicted within‐VAN (β = −.11, *p* = .03) and within‐DAN (β = −.12, *p* = .03) connectivity. Finally, the regression parameter for agreeableness significantly negatively predicted within‐DAN connectivity (β = −.12, *p* = .02). There were no significant relationships between within‐network connectivity in any of the above networks and extraversion or conscientiousness. The pattern of association did not change when re‐analyzing the data excluding education and IQ as covariates. Despite the significant results observed, none of them survived Bonferroni correction for multiple comparisons.

**Table 2:** Models reflecting the effects of personality traits on within‐network connectivity

Network	Number of observations/*R* ^2^	Independent variables	Unstandardized coefficients B	Standardized coefficients β	*p*‐value
Default mode	365 *R* ^2^ = .18	Age	.00	−.07	.20
		Sex	−.01	−.07	.09
		Education	.00	−.003	.94
		NART IQ	−.00	−.02	.91
		Openness	.01	.14	.02*
		Conscientiousness	.001	.01	.80
		Extraversion	−.006	−.08	.17
		Agreeableness	−.003	−.04	.45
		Neuroticism	.001	.01	.81
Fronto parietal control	365 *R* ^2^ = .11	Age	.00	−.05	.30
		Sex	−.007	−.04	.43
		Education	.00	−.002	.90
		NART IQ	.001	.08	.22
		Openness	−.004	−.05	.37
		Conscientiousness	.003	.04	.46
		Extraversion	.003	.04	.51
		Agreeableness	−.005	−.06	.30
		Neuroticism	−.001	−.01	.79
Ventral attention	365 *R* ^2^ = .15	Age	.00	.06	.24
		Sex	.005	.03	.53
		Education	.00	.01	.83
		NART IQ	−.001	−.06	.35
		Openness	.003	.03	.56
		Conscientiousness	.002	.02	.65
		Extraversion	.00	.006	.91
		Agreeableness	−.006	−.06	.24
		Neuroticism	−.01	−.11	.03*
Cingulo‐Opercular Control	365 *R* ^2^ = .29	Age	−.001	−.25	<.001*
		Sex	−.005	−.02	.59
		Education	.02	.06	.31
		NART IQ	.00	−.03	.57
		Openness	.008	.08	.16
		Conscientiousness	−.003	−.03	.58
		Extraversion	.003	.03	.52
		Agreeableness	.00	.005	.93
		Neuroticism	.002	.02	.69
Dorsal Attention	365 *R* ^2^ = .33	Age	−.002	−.23	.001*
		Sex	−.01	−.04	.34
		Education	.006	.12	.03*
		NART IQ	−.002	−.16	.01*
		Openness	.008	.07	.23
		Conscientiousness	−.002	−.01	.78
		Extraversion	−.002	.02	.72
		Agreeableness	−.01	−.12	.03*
		Neuroticism	−.01	−.12	.02*
Salience	365 *R* ^2^ = .14	Age	.00	−.10	.07
		Sex	.003	.01	.77
		Education	−.002	−.04	.44
		NART IQ	−.00	−.006	.93
		Openness	.006	.07	.23
		Conscientiousness	−.003	−.04	.47
		Extraversion	−.001	−.007	.90
		Agreeableness	−.003	−.04	.51
		Neuroticism	−.004	−.04	.41

* Significant *p*‐values (<.05). Models were run separately for each network.

### 3.3 Age moderation of personality–connectivity associations

**Figure 2:**
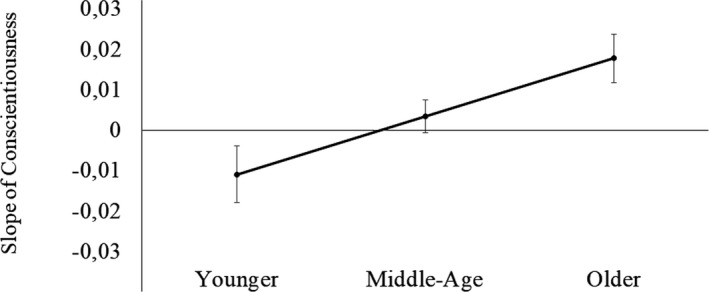
Age moderation of the effect of conscientiousness on FPC network connectivity. *Note:* Graph represents conscientiousness–FPC network within connectivity as a function of age. X‐axis represents age group; y‐axis represents beta values. Values above zero represent positive associations and below zero represent negative associations. Error bars = standard error; FPC = frontoparietal control network

**Figure 3:**
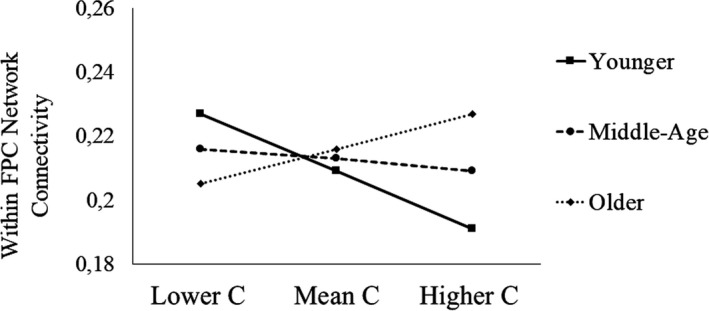
Conditional effects of age moderation. *Note:* FPC = frontoparietal control network; C = conscientiousness. Younger = −1 *SD*; Middle Age = mean age; Older = +1 *SD*

Age moderated the association between conscientiousness and within‐FPC‐network connectivity, indicating that this association was stronger for older adults (β = .17, *p* = .001; Table 3, Figure 2). To better clarify this interaction, we tested conditional effects of age moderation. We found that higher conscientiousness was associated with greater connectivity in the FPC network only for older adults (*p* = .004), while this relationship was not significant for middle‐age (*p* = .44) or younger adults (*p* = .07), although a trend was observed for the younger group (Figure 3).

**Table 3:** Model reflecting age moderation of conscientiousness on FPC within‐network connectivity

Network	Number of observations/*R* ^2^	Independent variables	Unstandardized coefficients B	Standardized coefficients β	*p*‐value
Fronto parietal Control	365 *R* ^2^ = .20	Age	−.00	−.01	.71
		Sex	−.006	−.04	.44
		Openness	−.001	−.01	.80
		Conscientiousness	.003	.03	.48
		Extraversion	.003	.03	.50
		Agreeableness	−.003	−.04	.46
		Neuroticism	.00	−.005	.93
		Age*Conscient	.001	.17	.001*

* Significant *p*‐values (<.05). Conscient = Conscientiousness.

### 3.4 Analysis considering scan length

Due to the two different scan lengths in our study and the fact that previous work has shown that scan length has a significant effect on functional connectivity metrics (Birn et al., 2013), we reran the models testing whether each of above significant associations mentioned above was moderated by scan length. Results showed that none of the significant effects were moderated by scan length. Regarding Model 1, scan length did not moderate the association between openness and DMN (*p* = .67), neuroticism and VAN (*p* = .38), neuroticism and DAN (*p* = .21), and agreeableness and DAN (*p* = .65). In Model 2, scan length did not moderate the age by conscientiousness interaction, although a trend was observed (*p* = .05).

## 4 DISCUSSION

Results from this study show associations between major dimensions of personality, as characterized by the FFM/Big Five (McCrae & Costa, 2004), and RSFC in predefined brain networks across the adult lifespan. Specifically, we found that higher levels of openness were associated with stronger within‐DMN connectivity, higher levels of neuroticism were associated with weaker within‐VAN and DAN connectivity, and higher levels of agreeableness were associated with weaker within‐VAN connectivity. In addition, age moderated the relationship between conscientiousness and FPN connectivity, such that this positive relationship was significant for older adults, but not for younger and middle‐aged adults.

Our results are in line with previous findings that openness implicates the DMN. For instance, others have found that openness predicted the global efficiency of a functional network comprised of DMN nodes, and, similar to our findings, this result remained significant after controlling for intelligence, age, sex, and other personality variables (Beaty et al., 2016). In addition, openness has been found to be positively associated with functional connectivity between DMN hubs and regions and networks associated with cognitive control (Adelstein et al., 2011; Beaty et al., 2018). Given the fact that people with higher openness/intellect tend to be imaginative, curious, innovative, and creative (Allen & DeYoung, 2016), we expected that the DMN would be a relevant substrate of this trait. The DMN has been reported to reflect spontaneous and self‐generated cognitive processes such as mental imagery, creative cognition, future thinking, autobiographical memory retrieval, theory of mind, mental scene construction, daydreaming, and mind‐wandering (Andrews‐Hanna, Smallwood, & Spreng, 2014; Fox, Spreng, Ellamil, Andrews‐Hanna, & Christoff, 2015; Pearson, 2019; Stawarczyk & D'Argembeau, 2015).

Although it remains to be clarified which regions of the DMN are more critically involved in the expression of openness, previous findings show that parietal regions of DMN may have a particular role in openness (Sampaio, Soares, Coutinho, Sousa, & Goncalves, 2014). This evidence is in line with a longitudinal finding that individuals with higher openness have a slower loss of gray matter volume in the right inferior parietal lobule, a DMN hub, and a region involved in creativity and working memory (WM; Taki et al., 2013). In addition, others have investigated the hypothesis that dopamine is part of the biological substrate of openness/intellect and has been associated with curiosity, exploratory behavior, and WM (Allen & DeYoung, 2016). For instance, Passamonti et al. (2015) showed that openness/intellect positively predicted functional connectivity between midbrain regions (from which dopaminergic neurons project, such as substantia nigra and ventral tegmental area) and the dorsolateral prefrontal cortex, a critical area for WM, in line with findings that openness is associated with WM (DeYoung, Peterson, & Higgins, 2005; DeYoung, Shamosh, Green, Braver, & Gray, 2009). Moreover, DMN–openness associations may underlie the evidence that openness is positively linked to several cognitive abilities, regardless of age (e.g., memory, fluid and crystallized intelligence, verbal fluency), as observed in studies including young, middle‐aged, and older adults (Graham & Lachman, 2014; Soubelet & Salthouse, 2011).

In our study, as anticipated, we did not find associations between extraversion and brain networks, similar to previous connectivity studies based on whole‐brain analysis (Dubois et al., 2018; Toschi et al., 2018). Neuroticism was negatively associated with within‐network functional connectivity, as previous reports (Nostro et al., 2018; Servaas et al., 2015). This negative association was observed in the VAN and DAN, both reflecting systems with specialized nodes promoting specific processes for attentional control. It has been proposed that the DAN mediates top‐down guided voluntary attention to locations or features allocation, whereas the VAN is involved in detecting unattended or unexpected stimuli and triggering shifts of attention (Corbetta & Shulman, 2002; Vossel et al., 2014). The fact that neuroticism was negatively linked to connectivity in both attention‐related networks may be connected to the “mental noise hypothesis,” indicating that higher neuroticism may increase mental noise, contributing to variability of cognitive performance and attention control deficits (Bredemeier et al., 2011; Robinson & Tamir, 2005; Robison et al., 2017). In addition, it has been hypothesized that higher neuroticism is linked with emotion dysregulation (Allen & DeYoung, 2016). Evidence in support of this view indicates that neuroticism is associated with reduced functional connectivity between the amygdala and several regions (e.g., dorsomedial prefrontal cortex, middle temporal gyrus and temporal pole; Adelstein et al., 2011; Aghajani et al., 2014). The fact that we observed reduced connectivity in the VAN may support the above hypothesis, since the VAN has been also implicated in both attentional control and emotion regulation (Viviani, 2013). In contrast to these previous findings, we did not observe any association between neuroticism and FPN or DMN networks (Servaas et al., 2015), and networks also implicated in psychiatric conditions (e.g., depression and anxiety), executive functions, and cognitive control and emotional regulation (Sylvester et al., 2012; Yan et al., 2019; Yao et al., 2019).

We found a negative association between agreeableness and within‐DAN connectivity, which has not been reported previously, and therefore not hypothesized, in the current study. Resting‐state seed‐based studies have reported that agreeableness is positively associated with functional connectivity among some hubs of the DMN (Adelstein et al., 2011; Sampaio et al., 2014); however, there is no association between this trait and RSFC in studies using a whole‐brain approach (Dubois et al., 2018; Toschi et al., 2018). Likewise, there are several reports on the lack of association between agreeableness and regional brain volumes (Bjornebekk et al., 2013; Liu et al., 2013), and cognitive performance (Graham & Lachman, 2014; Soubelet & Salthouse, 2011). Our results reinforce the idea that aggressive behavior (e.g., lower agreeableness) is linked to brain networks relevant for cognitive/attention control (Wong et al., 2019), although more evidence is necessary to better understand this relationship.

Contrary to our hypothesis, conscientiousness was not associated with networks relevant to cognitive control and goal priority (e.g., CON, VAN, and SAN; Rueter et al., 2018), but it was associated with FPN, considered a critical substrate of conscientiousness (Allen & DeYoung, 2016; Toschi et al., 2018). The FPN has been linked to top‐down cognitive control, particularly initiating and adjusting cognitive control (Dosenbach, Fair, Cohen, Schlaggar, & Petersen, 2008; Zanto & Gazzaley, 2013), reflecting skills endorsed by more conscientious individuals (e.g., those who are goal‐oriented, self‐disciplined, persistent, and able to suppress disruptive impulses). Nevertheless, we found that age moderated the association between FPN and conscientiousness, which was significant only in older participants. Our data add a unique contribution since we showed that the conscientiousness–FPN association increases as a function of age and may be particularly relevant for older adults. To the best of our knowledge, previous studies have not specifically analyzed relationships between brain connectivity and conscientiousness in older adults, since in many cases previous studies have systematically excluded older individuals (Allen & DeYoung, 2016; Passamonti et al., 2019; Rueter et al., 2018; Toschi et al., 2018). Although exploratory, our findings suggest that conscientiousness may be a protective resource for brain aging, being associated with higher within‐FPN connectivity, which is otherwise expected to decrease as a function of age (Campbell, Grady, Ng, & Hasher, 2012). Our result is in line with several observations that show conscientiousness as a predictor for academic or occupational success, healthy lifestyle, reduced cognitive decline, and longevity (Bogg & Roberts, 2013; Costa, Weiss, Duberstein, Friedman, & Siegler, 2014; Hock et al., 2014; Noftle & Robins, 2007; Ozer & Benet‐Martínez, 2006; Sutin & Terracciano, 2016).

In addition, we observed that within‐network connectivity metrics were more consistently associated with personality than IQ, which showed small and typically nonsignificant effects on brain connectivity (expect for DAN). This observation is similar to a study that found that openness had a greater effect than IQ on DMN (Beaty et al., 2016). Despite that, individual differences in intelligence have been related to changes in RSFC in neural networks broadly involved in self‐referential mental activity (e.g., DMN), attentional control processes (e.g., DAN and VAN), executive functions (e.g., FPN), and task‐set maintenance (e.g., CON; Hearne, Mattingley, & Cocchi, 2016).

The present work has as few limitations worth noting. Although our sample size is one of the largest in this area of research, we cannot rule out that our modest sample size may limit interpretation of our findings. In addition, the interpretation of the findings should be considered carefully due to the exploratory nature of our study and since results did not survive correction for multiple comparisons. The fact that we used a cross‐sectional design to investigate personality–connectivity associations does not allow us to infer causality or exclude cohort effects. Longitudinal design would have the potential to provide greater clarity on the current findings. In addition, personality data were examined only at the trait‐level, and not the subfactor level (e.g., facets), since the IPIP‐50 does not provide a validated facet‐level structure. Another potential limitation concerns the relatively shorter scanning duration (5 min) in part of the sample (*n* = 124) compared to other resting protocol (9.5 min) performed from most part of the participants (*n* = 241). Nevertheless, we did not find that scan length moderated any of our key results, which also remained consistent when replicating the analyses considering scan length as a covariate in the regression models Although unlikely, the difference in scan length could have had undetected effects on some of the connectivity metrics analyzed. In addition, the utilization of an externally derived network parcellation scheme for network assignment (Power et al., 2011) may be a limitation for the present study. Although previous studies have used network parcellation schemes derived from participants’ optimal network organization and cross‐registered these networks with nodal assignments in the Power et al. (2011) network taxonomy (Chan et al., 2014; Geerligs et al., 2015), this methodology is difficult to reproduce results in an external dataset due to differences in network structure in different samples. Considering this issue, we followed previous studies that similarly utilized the Power et al. (2011) taxonomy to define network structure/organization, indicating that this approach may be appropriate to estimate a plausible network structure that is not biased by participants in the sample (Song et al., 2014; Varangis, Habeck, et al., 2019; Varangis, Razlighi, Habeck, Fisher, & Stern, 2019). Lastly, it is relevant to highlight that some of the inconsistencies between our findings and those from other studies may be associated with differences in personality instruments, connectivity parcellation schemes adopted, and connectivity metrics chosen for analysis.

In summary, our findings extend those of previous studies showing associations among FFM/Big Five personality traits and within‐networks connectivity. We found robust and specific associations between openness and DMN, conscientiousness and FPN, neuroticism and attention networks (VAN and DAN), and agreeableness and DAN. Importantly, we added the unique contribution that age may be a relevant moderator of these personality–connectivity relationships, and future studies should include a wider age range when examining these associations across the adult lifespan. Our results contribute to understanding specific personality profiles that may be protective against different aspects of brain aging.

## CONFLICT OF INTERESTS

The author(s) declared no potential conflicts of interest with respect to the research, authorship, and/or publication of this article.

## Data Availability

The data that support the findings of this study are available from the corresponding author upon reasonable request.
